# *PHYTOCHROME B* and *HISTONE DEACETYLASE 6* Control Light-Induced Chromatin Compaction in *Arabidopsis thaliana*

**DOI:** 10.1371/journal.pgen.1000638

**Published:** 2009-09-04

**Authors:** Federico Tessadori, Martijn van Zanten, Penka Pavlova, Rachel Clifton, Frédéric Pontvianne, L. Basten Snoek, Frank F. Millenaar, Roeland Kees Schulkes, Roel van Driel, Laurentius A. C. J. Voesenek, Charles Spillane, Craig S. Pikaard, Paul Fransz, Anton J. M. Peeters

**Affiliations:** 1 Nuclear Organization Group, Swammerdam Institute for Life Sciences, University of Amsterdam, Amsterdam, The Netherlands; 2 Plant Ecophysiology, Institute of Environmental Biology, Utrecht University, Utrecht, The Netherlands; 3 Laboratory of Genetics, Wageningen University and Research Center, Wageningen, The Netherlands; 4 Genetics & Biotechnology Laboratory, Department of Biochemistry & Biosciences Institute, University College Cork, Cork, Republic of Ireland; 5 Biology Department, Washington University, St. Louis, Missouri, United States of America; Stanford University School of Medicine, United States of America

## Abstract

Natural genetic variation in *Arabidopsis thaliana* exists for many traits and often reflects acclimation to local environments. Studying natural variation has proven valuable in the characterization of phenotypic traits and, in particular, in identifying genetic factors controlling these traits. It has been previously shown that chromatin compaction changes during development and biotic stress. To gain more insight into the genetic control of chromatin compaction, we investigated the nuclear phenotype of 21 selected *Arabidopsis* accessions from different geographic origins and habitats. We show natural variation in chromatin compaction and demonstrate a positive correlation with latitude of geographic origin. The level of compaction appeared to be dependent on light intensity. A novel approach, combining Quantitative Trait Locus (QTL) mapping and microscopic examination, pointed at *PHYTOCHROME-B* (*PHYB*) and *HISTONE DEACETYLASE-6* (*HDA6*) as positive regulators of light-controlled chromatin compaction. Indeed, mutant analyses demonstrate that both factors affect global chromatin organization. HDA6, in addition, strongly promotes the light-mediated compaction of the Nucleolar Organizing Regions (NORs). The accession Cape Verde Islands-0 (Cvi-0), which shows sequence polymorphism in the *PHYB* gene and in the *HDA6* promotor, resembles the *hda6* mutant in having reduced chromatin compaction and decreased methylation levels of DNA and histone H3K9 at the NORs. We provide evidence that chromatin organization is controlled by light intensity. We propose that chromatin plasticity is associated with acclimation of *Arabidopsis* to its environment. The polymorphic alleles such as PHYB and HDA6 control this process.

## Author Summary

The habitat of the plant model species *Arabidopsis thaliana* can be found throughout the Northern hemisphere. As a consequence, individual populations have acclimated to a great diversity of environmental conditions. This is reflected by a wealth of natural genetic variation in many phenotypic traits. We utilized this natural variation via a novel approach, combining microscopic examination, quantitative genetics, and analysis of environmental parameters, to understand the regulation of nuclear chromatin compaction in leaf mesophyll cells. We show that the level of chromatin compaction among natural *Arabidopsis thaliana* accessions correlates with latitude of origin and depends on local light intensity. Our study provides evidence that the photoreceptor PHYTOCHROME-B (PHYB) and the histone modifier HISTONE DEACETYLASE 6 (HDA6) are positive regulators of global chromatin organization in a light-dependent manner. In addition, HDA6 specifically controls light-mediated chromatin compaction of the Nucleolar Organizing Regions (NORs). We propose that the observed light-controlled plasticity of chromatin plays a role in acclimation and survival of plants in their natural environment.

## Introduction

Plant phenotypes are the integrated result of developmental programs and plastic responses to the environment. *Arabidopsis thaliana* has a wide biogeographical distribution. Consequently, rich natural (genetic) variation exists among collected accessions [1]–[4], which are acclimated to environmental conditions in their local habitat. Utilization of this natural variation in functional studies has led to a better understanding of the molecular and physiological mechanisms of complex traits such as the acclimation to the light environment [5]–[11].

We recently observed variation in chromatin compaction during floral induction in three *Arabidopsis* accessions [12], suggesting the existence of natural genetic variation for chromatin organization. Chromatin folding is an essential process in eukaryotes, which provides differential accessibility of genes and regulatory elements along the linear DNA sequence. At the microscopical level different types of chromatin can be discerned depending on the condensation degree. For example, in *Arabidopsis* nuclei the chromosomes display highly condensed heterochromatin domains (chromocenters) and less condensed gene-rich euchromatin loops [13],[14]. The main component of chromocenters is repetitive DNA which includes long tandemly arranged DNA elements, such as satellite repeats, ribosomal-DNA (rDNA) genes and centromeric sequences. The chromocenters contain epigenetic markers for heterochromatin. Quantification of chromocenter size and intensity has been used to assess chromatin compaction in several studies [12], [14]–[17].

The ensemble of cytogenetically-defined functional parameters constitutes the nuclear phenotype, which is associated with specific transcriptional states [14],[18]. The different states of chromatin compaction are accompanied by specific epigenetic markers [19]. Methylation of both cytosine and histone H3 lysine 9 occurs in transcriptionally repressed areas, whereas methylation at histone H3K4 and histone acetylation mark regions of gene activity.

Several studies have indicated plasticity in chromatin compaction during development and upon interaction with the environment [14], [16], [17], [20]–[22] (reviewed in [23]). For example, heterochromatin levels rise during seedling establishment [17], and the heterochromatin content of young mesophyll cells is lower than in fully differentiated leaves [14]. In addition, Pavet and colleagues observed severe loosening of chromocenters and hypomethylation upon infection by *Pseudomonas syringae* pv. *Tomato* [22]. Recently, it was shown that formation of totipotent protoplasts coincided with a strong reduction of heterochromatin compaction [16].

Despite the increasing amount of data showing large-scale reorganization of chromatin domains, we still know very little about the genetic components controlling the plasticity of chromatin. Here, we demonstrate natural variation in chromatin organization in 21 *Arabidopsis* accessions, originating from different geographic origins. The level of chromatin compaction correlates with latitude of origin and depends on light intensity. We utilized natural variation in a quantitative genetic approach to identify loci affecting chromatin organization. We provide evidence that the photoreceptor *PHYTOCHROME-B* (PHYB) and the histone modifier *HISTONE DEACETYLASE 6* (HDA6) control light-dependent chromatin organization.

## Results

### Chromatin compaction correlates with geographic latitude of origin and local irradiation levels

**Figure 1:**
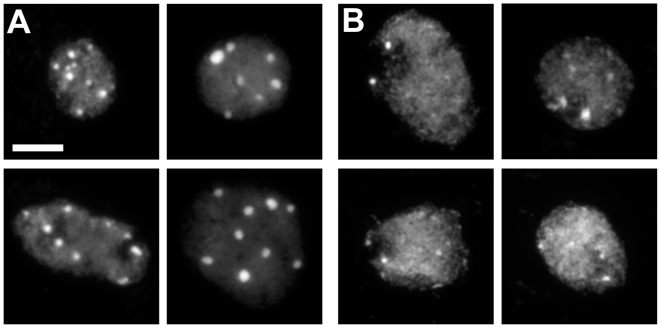
L*er* nuclei display high chromatin compaction and conspicuous chromocenters, in contrast to Cvi-0. Nuclei are counterstained with DAPI. (A) L*er* nuclei have 6–10 conspicuous, round chromocenters. (B) Cvi-0 nuclei have fewer and smaller chromocenters. Bar = 5 µm.

To study natural variation in chromatin compaction, we examined the chromocenter phenotypes of leaf mesophyll cells from 21 accessions originating from a wide variety of natural habitats. We observed large variation in chromocenter size and number between accessions (Figure 1). To quantify this variation, we used the heterochromatin index (HX; [12]), which is a measure of the fraction of nuclei with conspicuous chromocenters (i.e. the typical Landsberg *erecta* (L*er*) phenotype in Figure 1A) over the total number of nuclei. The variation in HX ranged from 0.19 (Cape Verde Islands; Cvi-0) to 0.92 (Kondara; Table S1).

**Figure 2:**
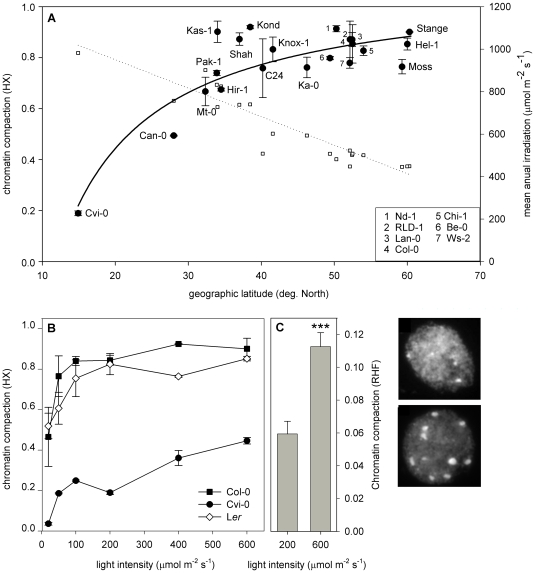
Chromatin compaction correlates with latitude of origin and light intensity levels. (A) Chromatin compaction correlates with latitude of origin. Left Y-axis represents chromatin compaction (HX) of plants grown at a light intensity of 200 µmol m^−2^ s^−1^ in short-day photoperiod (closed round symbols), and right Y-axis represents mean annual irradiation (open squares), on the geographic latitude of collection sites. The best-fitted curves are shown (HX; closed line, mean annual irradiation; dotted line). Inset shows abbreviations of the clustered accessions depicted above in the graph. n≥2. (B) Chromatin compaction of Col-0 (black squares), L*er* (white diamonds), and Cvi-0 plants (black circles), grown in different light intensities. n≥2. (C) Relative Heterochromatin Fraction (RHF) of Cvi-0 grown in different light intensities in short-day conditions. n≥30, *** p<0.001 significance value compared to standard conditions (200 µmol m^−2^ s^−1^). Error bars represent SE in al cases. Images show nuclei of Cvi-0 plants grown at 200 µmol m^−2^ s^−1^ (left) and 600 µmol m^−2^ s^−1^ (right).

Because the 21 accessions originate from different geographic locations (Figure 2A, Table S2), we tested if geographic origin correlated with chromatin compaction. We found a significant (p<0.001) inverse correlation (r^2^ = 0.76) between HX and geographical latitude of origin (Figure 2A). Longitude and altitude did not display a significant correlation. Subsequently, we analyzed if local environmental conditions correlated with the chromatin phenotypes. We used mean annual climate parameter data acquired over a 30 years period ([24]; Table S2). Stepwise removing of the least-significant parameters from a multiple-regression analysis, revealed that mean annual irradiation (p<0.001) and annual amount of wet-days (p = 0.01) may explain the geographic variation in HX. These parameters also correlated best with latitude (Table S2). Although day length is a clear latitude-dependent parameter that affects e.g. flowering time [25],[26] and circadian period [27], it does not have impact on heterochromatin compaction (data not shown). Apparently, the photon flux density (light intensity) rather than daily quantum input influences chromatin compaction.

To test if light intensity directly influences chromatin organization, we examined the accession with the lowest HX, the sub-tropical accession Cvi-0, together with the commonly used Central-European laboratory accessions, Columbia-0 (Col-0) and L*er* at different light intensities. Below 50 µmol m^−2^ s^−1^, these accessions showed a lower HX than under standard conditions (Figure 2B). However, above 100 µmol m^−2^ s^−1^ for Col-0 and 200 µmol m^−2^ s^−1^ for L*er*, the HX reached a plateau of 0.8–0.9. Strikingly, the HX of Cvi-0 increased over the entire range of light intensities used. These results confirm that chromocenter compaction depends on light intensity.

In contrast to HX, which assesses variation in chromatin compaction in a population of nuclei, the Relative Heterochromatin Fraction (RHF) reflects the chromocenter compaction per nucleus [14],[15]. The positive correlation between light intensity and chromatin compaction in Cvi-0 was confirmed by RHF measurements (Figure 2C). Together, these data indicate that below certain irradiation intensities, light becomes a limiting factor for chromatin compaction. This is reflected by a decrease of the nuclear fraction with conspicuous chromocenters resulting in lower HX. The threshold varies between different accessions and is above 600 µmol m^−2^ s^−1^ for Cvi-0.

### Altered localization of repeats and epigenetic markers in nuclei of Cvi-0

**Figure 3:**
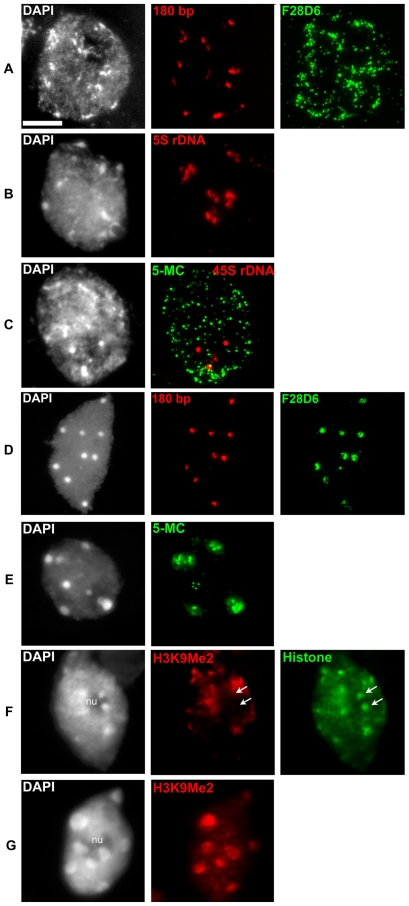
Cytogenetic characterization of Cvi-0. FISH signals for the centromeric 180 bp [(A), red] and the subtelomeric 45S rDNA repeats [(C), red] are compact and located at chromocenters. Signals for the pericentromeric sequences 5S rDNA [(B), red] and transposon-rich BAC F28D6 [(A), green] are dispersed and outside heterochromatic regions. For comparison, in Col-0 both the centromeric 180 bp [(D), red] and BAC F28D6 [(D), green] are compact and located at the chromocenters. Immunolabeling of 5-Methylcytosine [(C), 5-MC, green] reveals a dispersed pattern in Cvi-0 compared to the clustered immunosignals in Col-0 [(E), 5-MC, green]. Note the absence of 5-MC signal on the Cvi-0 45S rDNA sequences [(C), red]. Immunolabeling of H3K9Me2 [(F), red] reveals the absence of this epigenetic mark on NOR chromocenters at the periphery of the nucleolus [(F), arrows] in Cvi-0, while all chromocenters are marked in Col-0 [(G), red]. Histone immunolabeling on Cvi-0 [(F), green] was carried out as control for histones. Each nucleus was counterstained with DAPI (first column). nu: nucleolus; Bar = 5 µm.

Chromatin compaction at chromocenters involves the condensation of repetitive DNA sequences such as the centromeric repeats, ribosomal genes and transposable elements [18]. To find out which sequences remain in the reduced chromocenters of Cvi-0, we applied Fluorescence *In Situ* Hybridization (FISH) using the 180 bp centromere repeat, 5S rDNA, 45S rDNA and the BAC F28D6 (Figure 3A–3C). The latter contains many dispersed repeats such as transposons, which are predominant in pericentric regions (Figure S1). The 180 bp centromeric tandem repeats and 45S rDNA subtelomeric region displayed condensed signals at chromocenters (Figure 3A and 3C), similar to Col-0 and L*er* [12],[16]. In contrast, 5S rDNA and BAC F28D6 signals showed a dispersed pattern (Figure 3A and 3B). This suggests that the loss of chromatin compaction in Cvi-0 is caused by dislocation of pericentric repeats away from the chromocenters, comparable to the situation observed in gene silencing mutants such as *ddm1* and *met1* [15].

Since chromocenters contain most epigenetic markers for gene silencing [18],[28], we examined if the epigenetic patterns were affected in Cvi-0 nuclei (Figure 3C–3F). Immunolabeling revealed that 5-Methylcytosine (5-MC) is concentrated in chromocenters of Col-0, whereas in Cvi-0 the 5-MC label is dispersed over the entire nucleus (Figure 3C and 3D), similar to the pericentric repeats. Even the Nucleolar Organizing Region (NOR) chromocenters are hypomethylated. Hence, the dispersed 5-MC pattern, supports the FISH results, that low chromatin compaction at chromocenters in Cvi-0 is due to dispersed repeat regions. Immunostaining of H3K9me2 showed moderate dispersion and a diffuse signal at chromocenters in Cvi-0. Interestingly, both 5-MC and H3K9me2 labeling in Cvi-0 was markedly reduced in the large chromocenters that flank the nucleolus (Figure 3C and 3E). These chromocenters contain the rDNA genes (NOR) of chromosomes 2 and 4 [13],[29],[30]. This is not the case for Col-0 (Figure 3D and 3F), where all chromocenters show distinct H3K9me2 and 5-MC signals. Apparently, the ribosomal genes of Cvi-0 have decreased levels of both 5-MC and H3K9me2.

### QTL mapping reveals three loci controlling chromatin compaction

We applied Quantitative Trait Locus (QTL) analysis to map loci controlling light-dependent chromatin compaction. For this aim, RHF was measured in 47 selected L*er* x Cvi-0 Recombinant Inbred Lines (RILs) [31]. We based our analysis on RHF, because this is a composite quantitative trait that combines nuclear and individual chromocenter size and intensity, which is a strong valuation of chromatin compaction in each line.

**Figure 4:**
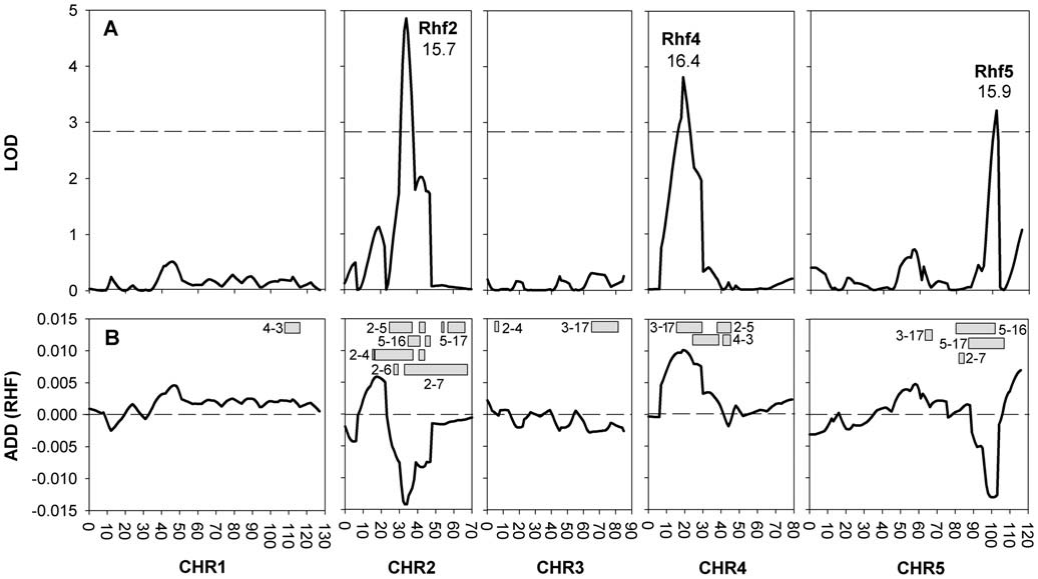
QTL analysis reveals three loci explaining the RHF. (A) QTL-LOD profile of RHF per chromosome (CHR). The dashed line marks the 95% confidence threshold at LOD 2.89. Percentage explained variance and QTL names are given near each QTL peak. (B) Additive effect of the Cvi-0 allele compared to the population average. Boxes schematically show the positions of the Cvi-0 introgressions of the NILs used to confirm the QTLs. Numbers depicted are the LCN line-numbers. The average map position of the flanking L*er* marker and the border Cvi-0 marker are depicted as cross-over position.

Heterozygous lines of L*er* x Cvi-0 and Cvi-0 x L*er* crosses revealed a low RHF, indicating that the Cvi-0 RHF phenotype is dominant. The broad sense heritability (H^2^) was 0.4 indicating that 40% of the total variation is explained by genetic differences between the RILs. Three QTLs, designated Rhf2, Rhf4 and Rhf5, were above the permutation calculated Likelihood Of Odds (LOD) threshold [32] of 2.89 (Figure 4A). Together, these QTLs explained 53.2% of the variation in RHF in the population. No epistasis could be detected between the QTLs (data not shown).

Since L*er* has a higher RHF than Cvi-0, QTLs with a negative allelic effect for Cvi-0 were anticipated. However, Rhf4 had a positive Cvi-0 effect implying the existence of both positive and negative molecular regulators contributing to the average RHF (Figure 4B).

**Figure 5:**
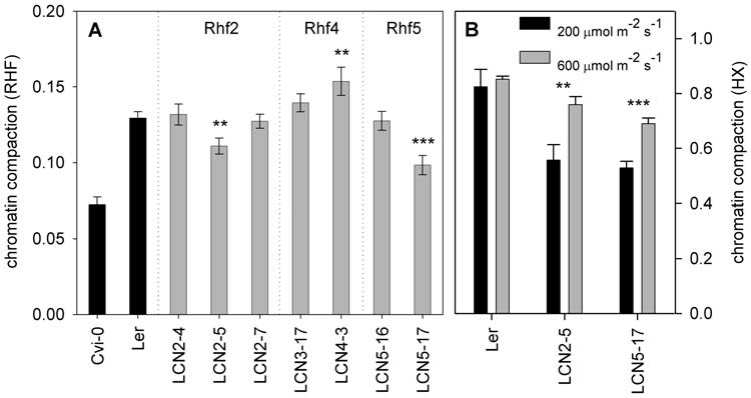
Near Isogenic Lines confirm the QTL positions and effects. (A) RHF of the NILs with a Cvi-0 introgression in the L*er* genetic background at the Rhf QTL loci, in control light conditions. The parents used to generate the NILs are in black. NILs are in gray. QTL names are depicted above the NILs. n>13 nuclei. (B) HX (n≥2) of LCN2-5 and 5-17 at 200- (black bars) and 600 µmol m^−2^ s^−1^ (gray); Error bars represent SE in al cases. ** p<0.01; *** p<0.001 significance value compared to L*er* (A) or compared between plants of one genotype grown in 200 µmol m^−2^ s^−1^ compared to the same genotype grown in 600 µmol m^−2^ s^−1^ (B).

To confirm the location and effect of the putative QTLs, we measured the RHF of Near-Isogenic Lines (NIL) covering and flanking the QTL positions. These NILs contain small Cvi-0 introgressions in the isogenic L*er* genetic background [33],[34] (Figure 4B). For each QTL, we found one NIL with a predicted and significantly different RHF effect (Figure 5A). The introgression region in this NIL thus contains a Cvi-0 allele that contributes to the total observed low Cvi-0 RHF. Rhf2 is explained by NIL LCN2-5 (introgression on chromosome 2 between 22 and 27 cM). Surprisingly, LCN2-4 was not significantly different from L*er* (Figure 5A), most likely due to the linked opposite QTL (Figure 4) at this locus which may repress the LCN2-4 phenotype. Alternatively, (flanking) positive, additive Cvi-0 alleles that are not detectable by QTL analysis may cause this effect. The Rhf4 region is explained by LCN4-3 and could be assigned, using other NILs, to 35 cM and 50 cM. Similarly, the region for Rhf5 was explained by LCN5-17 and could be restricted to only 3 cM (between 107 and 110 cM). Subsequently, we measured the HX of the two NILs with reduced RHF (LCN2-5 and LCN5-17). Both NILs had a reduced HX compared to L*er*, confirming the observations with RHF (Figure 5B). When these lines were grown at 600 µmol m^−2^ s^−1^ we observed significantly increased HX values in both lines, compared to plants grown at 200 µmol m^−2^ s^−1^ (p<0.001 for LCN5-17 and p<0.01 for LCN2-5), while the HX of L*er* remained unchanged. These data indicate that *Arabidopsis* chromosomes 2 and 5 contain loci (within the introgression of LCN2-5 and LCN5-17, respectively) that are segregating between L*er* and Cvi-0 accessions and influence chromatin compaction in a light dependent manner.

### PHYTOCHROME-B and HISTONE DEACETYLASE 6 are involved in light-mediated chromatin compaction

**Figure 6:**
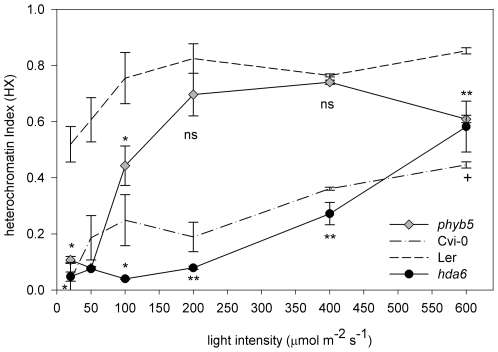
PHYTOCHROME-B and HISTONE DEACETYLASE-6 are positive regulators of light-dependent chromatin compaction. Heterochromatin index of *phyB5* (gray diamonds), *hda6 sil1/not1* (black circles), Cvi-0 (dash-dotted line), and L*er* (dashed line) grown in different light intensities. n≥2. *** p<0.001; ** p<0.01; * p<0.05; ^+^ p<0.1; ns = non significant; significance value compared to L*er*. Error bars represent SE in al cases.

Within the small introgression regions of LCN2-5 and LCN5-17 that contributed to the low RHF of Cvi-0, we selected the photoreceptor *PHYTOCHROME-B* (*PHYB*), and *HISTONE DEACETYLASE-6* (*HDA6*) for further study, based on their annotation as light perception and chromatin component (Table S4, Table S5, Table S6). Although the chromatin remodeler *DDM1* is also located on the introgression region of Rhf5, we did not consider this gene, since Cvi-0 has normal overall DNA methylation levels [35]. Both *hda6* (*hda6 sil1/not*; [36]) and *phyB5* [37] mutants showed a significantly reduced HX and RHF compared to L*er* (Figure 6; Figure S2), indicating that these candidate genes affect chromatin compaction. Moreover, inactivating PHYB in a non-invasive manner, by application of low red-to-far red ratio light, mimicking natural canopy shade, did also result in a reduced chromatin compaction (HX; 0.34±0.13), comparable to the *phyB5* effect. A low chromatin compaction has been described before for *hda6* [18],[38], but not for *phyB*.

Next, we performed complementation analysis [39],[40] on F_1_ crosses between mutant and wild types. In all cases, F_1_ lines from crosses with Cvi-0 showed low RHF levels and were statistically identical to Cvi-0, but never to L*er* (Figure S2), indicating dominance of the Cvi allele(s). However, heterozygous F_1_ plants of crosses between *phyB* or *hda6* mutants and L*er*, in contrast to Cvi-0, showed intermediate levels of RHF (Figure S2; significantly different from both Cvi-0 and L*er*), suggesting that none of these alleles is dominant.

**Figure 7:**
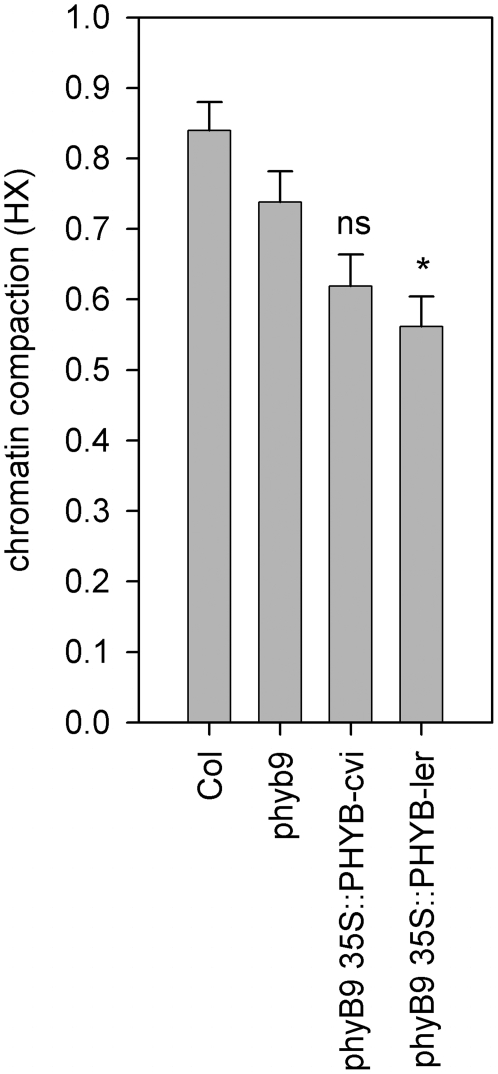
Ectopic expression of the L*er* PHYB allele, but not the Cvi-0 allele, reduces chromatin compaction. Heterochromatin index of *phyb9* mutant in the Col genetic background, complemented with gain-of-function PHYB alleles from Cvi-0 or L*er* [41] at 200 µmol m^−2^ s^−1^ in short-day photoperiod. Significance levels are compared to *phyB9* values; * p<0.05; ns = non significant. Error bars represent SE in al cases.

To assay the light dependency of the chromatin compaction phenotypes in *hda6* and *phyB5*, both lines were examined under different light intensities. In general, the mutants displayed a lower HX than wild type L*er* (Figure 6). Similar results were obtained using *phyB9* in the Col-0 background (Figure 7). At higher light intensities, the HX increased drastically in both mutants, illustrating the interaction between light intensity and chromatin compaction levels. Interestingly, we observed differences in the response to light intensity between *hda6* and *phyB5*. Up to 200 µmol m^−2^ s^−1^, the chromatin compaction in *phyB*5 increased rapidly to values close to L*er*, whereas in *hda6* the chromatin compaction increased steadily after 200 µmol m^−2^ s^−1^, reminiscent of the pattern of Cvi-0. Thus, PHYB predominantly acts at low light intensity (<200 µmol m^−2^ s^−1^), whereas HDA6 plays a role throughout the light intensity range. This demonstrates a novel function of the histone modifier HDA6. The data indicate that PHYB and HDA6 are positive controllers of light intensity-mediated chromatin compaction. However, we cannot exclude that other genes underlying the QTLs might also influence chromatin compaction.

### Segregating *PHYB* alleles contributes to the low chromatin compaction levels of Cvi-0

Recently, polymorphisms have been identified in *PHYB* between L*er* and Cvi [41]. Ectopic overexpression of PHYB resulted in increased light sensitivity. However, overexpression of the Cvi allele sensitized the plant less than the L*er* allele, indicating that the Cvi allele is less able to confer sensitivity to light. We examined chromatin compaction in *phyB9* complemented with *35S*::*PHYB*-Cvi and *35S*::*PHYB*-L*er*, to test if the same allelic variation accounts for variation in chromatin compaction. Both showed reduced chromatin compaction at 200 µmol m^−2^ s^−1^, indicating that besides light reduction, increased light sensitivity also results in reduction of chromatin compaction (Figure 7). The *35S*::*PHYB*-Cvi lines however, displayed a non-significant reduction, whereas the L*er* allele conferred a significant (p<0.05) reduction. This confirms the reduced light sensitivity of the Cvi allele as compared to L*er*.

The direct comparison of the data of the *35S::PHYB-*Cvi and *35S::PHYB-*L*er* lines did not result in significant differences due to very small differences in effects between the alleles. This is in accordance with Filiault and co-workers [41] and supports their conclusion that the differences in effect of *PHYB-*Cvi *PHYB-*L*er* are only small. This is also in agreement with the small additional effect of the Rhf2 QTL (Figure 4) and with the mild phenotype of NIL LCN2-5 (Figure 5). Therefore, we compared the effects of the transgenic lines with the null mutant *phyb9*, in the Col-0 genetic background independently, to increase the phenotypic-effect window. This did result in a significant difference between *phyB9* and *PHYB-*L*er*, but not between *phyB9* and *PHYB-*Cvi, which strongly suggests that allelic variation in *PHYB* at least contributes to Rhf2 QTL. Alternatively, these effects can be explained by (differential) interactions between the alleles and factors in the Col-0 background [41].

Among the polymorphisms in *PHYB* segregating between L*er* and Cvi, five are shared by Cvi and a clade of Spanish accessions [41]. At least two of these Spanish accessions; Ts-1 (HX = 0.58±0.01) and Se-0 (0.67±0.02) show a low chromatin compaction compared to other European accessions. Together, these data suggest that variation in the *PHYB* sequence contributes to variation in chromatin compaction and likely explains the Rhf2 QTL.

### Cvi differs from L*er* in *HDA6* sequence and resembles *hda6* mutant in chromatin compaction

**Figure 8:**
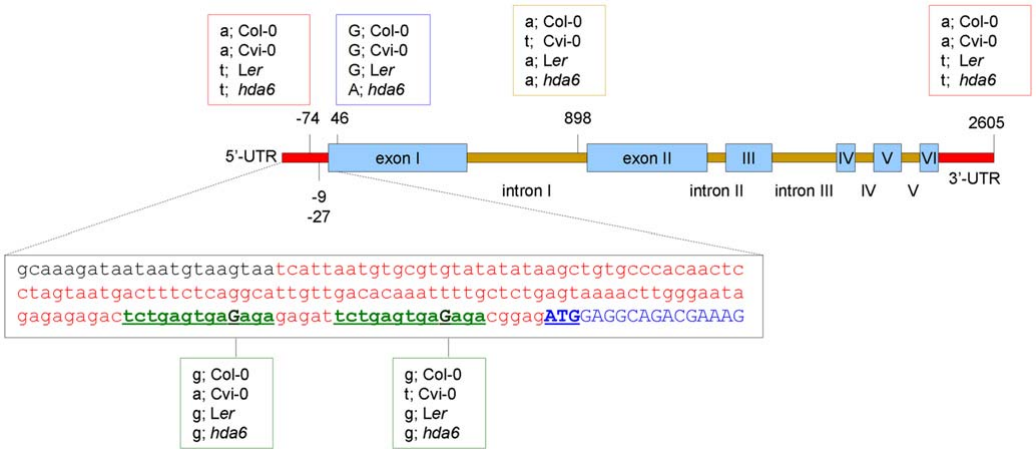
Cvi-0 has *HDA6* 5′-UTR polymorphisms compared to L*er* and Col-0. Schematic representation of the *HDA6* (At5g63110) genomic sequence alignment in; Col-0, Cvi-0, L*er*, and *hda6 sil1/not*. Untranslated regions (UTR; red), exons (blue boxes), and introns (orange bars) are shown along with the identified polymorphisms in bp relative to the ATG start codon. *Hda6* represents the *hda6 sil1/not* mutant. Sequences were aligned using CLUSTAL W 2.0 multiple sequence alignment and the alignment produced using EMBL EBI tools. The zoom-in box shows the sequence of the *HDA6* 5′-UTR (red) and the start of the first exon (blue, start codon; bold/underlined). The small repeat sequences that harbor the polymorphism in Cvi-0 relative to Col-0 and L*er* are shown in green (underlined) and the polymorphism site is shown in dark green. Note a G/A nucleotide substitution at bp 47 in the *hda6 sil1/not* mutant, which results in a predicted amino acid change from glycine (G) to arginine (R) in the *hda6 sil1/not* allele, confirming the results of Probst et al. [56]. Polymorphisms did not influence histone deacetylase signatures (as found using the NASC genome browser and extracting motifs using the SPRINT Prints view) nor potential miRNA targets, from http://sundarlab.ucdavis.edu/cgi-bin/mirna/.

No sequence differences were found in the coding region of the *HDA6* alleles in L*er*, Cvi-0 and Col-0, except for the 5′-UTR and the first intron (Figure 8). An intriguing feature was the presence of two base substitutions, each at an identical position in a small 13 bp repeat sequence in the 5′-UTR region of *HDA6* in Cvi-0. Subsequently, we examined if different *HDA6* mRNA isoforms were produced in Cvi-0. Reverse Transcriptase-PCR analysis revealed no evidence for altered expression levels nor alternative splicing products (data not shown).

**Figure 9:**
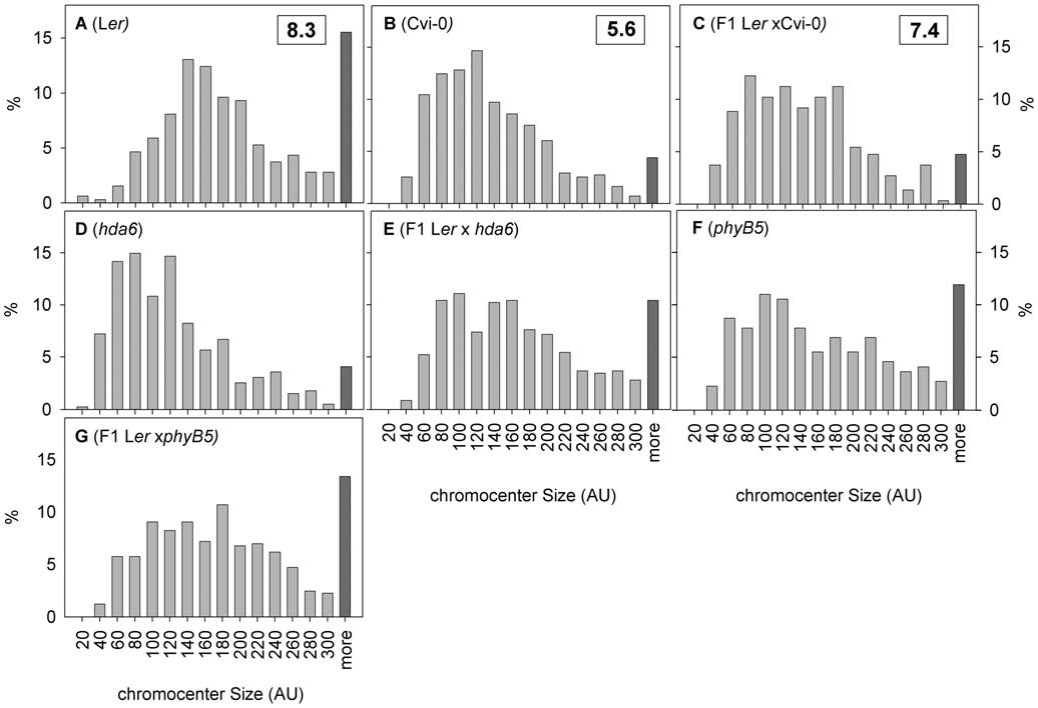
NOR chromocenter size is specifically affected in Cvi-0 and *hda6*. Distribution of size per individual chromocenter in Arbitrary Pixel Units (AU) of F1 plants derived from crosses between *hda6* and *phyB* mutants and their parental wild types. Boxed numbers indicate the average number of detectable chromocenters per nucleus. The highest class for both distributions is defined on a 10% cutoff.

To further investigate the cause of the difference in RHF between the Cvi-0 and L*er* heterozygotes, we dissected the RHF, which is a measure for the whole nucleus, into its components and examined the size of individual chromocenters (Figure 9). Small chromocenters are more frequently observed in Cvi-0 than in L*er* (Figure 9A and 9B). This is not due to chromocenter association in L*er*, since the average number of chromocenters per nucleus in L*er* (8.3) is higher than in Cvi-0 (5.7). Cvi-0 lacks the very large chromocenters (>300 area units) (Figure 9B), which contain the NORs [13]. The fraction of large NOR chromocenters increases at higher light intensity (Figure S3), suggesting that NOR formation is controlled by light.

Remarkably, *hda6* resembles Cvi-0 in having small NOR chromocenters (Figure 9D), suggesting that Cvi-0 has a malfunctional HDA6 allele. In contrast, the size of NOR chromocenters in *phyB* equals those in L*er* (Figure 9A and 9F), implying that chromatin compaction in the photoreceptor mutant is brought about by a different process compared to *hda6*.

Interestingly, the Cvi-0 phenotype with small NOR chromocenters, is dominant in the heterozygous L*er* x Cvi-0 (Figure 9C) whereas the phenotype for small NOR chromocenters is intermediate (Figure 9C vs. 9E) in the F_1_ L*er* x *hda6*. The added parental data (L*er* + *hda6*) strongly resembles the distribution of chromocenter size in the heterozygote (L*er* x *hda6*) (Figure S4). This indicates that half of the chromocenters in the heterozygotes have the wild type appearance and the other half have the *hda6* appearance. This is particularly noticeable for the NOR chromocenters. In contrast, the distribution patterns of Cvi + L*er* and the heterozygous L*er* x Cvi-0 do not superimpose, pointing to inheritance of chromocenter size.

## Discussion

### Natural variation in *Arabidopsis* chromatin compaction reflects latitudinal variation in light intensity

*Arabidopsis* is an excellent model plant to study natural variation [2]. Here, we demonstrate latitudinal variation in chromatin organization, measured in leaf mesophyll of 21 diverse accessions. Since all accessions were grown under identical conditions, the nuclear phenotype likely reflects the difference in environmental conditions between the growth chamber and the habitats of their origin. Light irradiation at the geographic origin sites turned out to be the best parameter for explaining the observed differences in chromatin compaction. Plants from habitats closer to the equator are exposed to higher light irradiation. Consequently, in the growth chambers these plants sense low light. The suggested correlation between light response and latitude is in accordance with the observations of Maloof et al. [9] who studied hypocotyl length and demonstrated a correlation between light sensitivity and latitude of origin. The authors concluded that accessions closer to the equator are less sensitive to light. Accordingly, a high frequency of the PHYTOCHROME-C haplotype, that compensates for lower light intensities at high latitudes, was found in Northern accessions [42]. Moreover, it is well known that flowering responses, that is a light controlled trait, correlate with latitude [43]. Apparently, natural variation in light sensitivity is reflected in clinal phenotypic variation. Hence, we consider chromatin compaction as a phenotype that reflects light sensitivity. This light-latitude correlation explains why in the most southern accession, Cvi-0, light intensity is a limiting factor for chromatin compaction up to at least 600 µmol m^−2^ s^−1^, whereas in the Central European accessions Col-0 and L*er* the threshold is found around 100 µmol m^−2^ s^−1^. We cannot exclude however, that other geographical parameters may have additional effects on chromatin organization. The absence of collected accessions at latitudes between Can-0 and Cvi-0 limited the statistical power. Nevertheless, to our knowledge, this is the first report of a correlation between clinal origin of a plant and large-scale nuclear organization.

### Genetic analysis reveals *PHYB* and *HDA6* as candidate genes for light-dependent chromatin compaction

Natural variation in chromatin organization was exploited to identify genes affecting light-dependent chromatin organization. We isolated and confirmed QTLs for chromatin compaction, together explaining 53% of the total variation in chromocenter compaction between L*er* and Cvi-0. There are several ways to identify potential polymorphisms that might explain QTLs. The standards for proving the molecular identity of QTLs, however, are somewhat subjective [44],[45]. Here, we reduced the number of the candidate genes using NILs. We then chose a candidate gene approach, which resulted in *PHYB* and *HDA6* as target genes. Both proved to be involved in light-dependent chromatin organization. However, we did not unambiguously show that allelic variation in the *PHYB* gene is causal for the QTL Rhf2, since the phenotypic trait is multigenic. This is a general difficulty in obtaining final causal proof for identified polymorphisms. If one of the segregating components is dominant, as in our case with Cvi-0 and chromatin compaction, then several direct approaches to identify the QTL (e.g. genetic complementation) are severely hampered. Also transgenic analysis by reciprocally transforming the candidate genes to the respective parents would probably not be informative due to the dominance effects. Consequently, we chose to analyse complemented *phyb9* mutants [41]. A potential drawback of such a transgenic approach is that variable expression levels of independent transformants may result in variable phenotypes. Therefore, we batched many independent transformants prior to analysis and provided indirect evidence that *PHYB* at least contributes to the QTL Rhf2. Unequivocally proving that an identified allele is causal for a mapped QTL for a phenotypic trait which involves multiple components, remains a serious problem. To conclusively prove the causality of *PHYB* for Rhf2, one should replace the *PHYB-*Cvi allele in LCN2-5 by the *PHYB-*L*er* allele. We expect that this fully rescues the reduced chromatin compaction phenotype. Ideally, the new allele should replace the endogenous allele in this experiment via homologous recombination. These gene-targeting systems are available in Drosophila [46] but remain very difficult in higher plants, although promising advances have been made in recent years [47],[48].

### Light-dependent chromatin compaction requires PHYB

PHYB was previously identified as a candidate gene explaining a light-related QTL in the same L*er* x Cvi-0 RIL population [7]. In addition, sequence polymorphisms in *PHYB* between L*er* and Cvi-1 has been reported to cause differences in light response [41]. A serine-to-threonine substitution in the PAS-A domain appeared to affect hypocotyl length. The same PAS-A allele is also present in a clade of Spanish accessions that show lower chromatin compaction compared to among others, Kondara and Kashmir-1. These accessions do not belong to the ‘Spanish’ clade, but originate from similar latitude, supporting the idea that the polymorphism in the PAS domain contributes to the difference in light-dependent chromatin compaction.

A similar polymorphism is present in the PAS-B domain where an isoleucine in Cvi-0 (Genbank IDs; AAW56575) replaces a serine in Col and L*er* (AAD08948, AAW56578). Both PAS-A and PAS-B polymorphisms involve serine, which is often subject to phosphorylation for functional conformational changes in phytochromes [49]. The PAS-A/B domain is required for import into the nucleus [50]. A serine mutation in the PAS A/B domain may disturb the translocation of PHYB into the nucleus. Once translocated into the nucleus PHYB can physically interact with CRYPTOCHROME2 (CRY2) in a light-dependent fashion [38]. Strikingly, CRY2 has been demonstrated to control chromatin condensation during the floral transition in a light-dependent manner [12]. These data indicate that Cvi-0 has aberrant PHYB activity which contributes to the reduced chromatin compaction.

### Cvi-0 resembles the *hda6* mutant for chromatin organization at the NOR

Similar to Cvi-0, the *hda6* mutant (*sil1/not*) displays a low chromocenter compaction that can be restored at higher light intensities. HDA6, which co-locates on QTL Rhf5, is a histone deacetylase, known to control a variety of biological processes such as flowering [51], regulation of transcription factors [52], transcriptional silencing of transgenes and repeats [53]–[58]. A major target of HDA6 activity is the rDNA repeat locus [56]. Arabidopsis *hda6* mutants display decondensation of NOR chromocenters and reduced rDNA methylation. Moreover, HDA6 is involved in nucleolar dominance [59],[60]. An RNAi-mediated knock-down of HDA6 in *Arabidopsis suecica*, the natural hybrid between *Arabidopsis thaliana* and *Arabidopsis arenosa*, resulted in reactivation of the silent *A. thaliana* rDNA genes and decondensation of its NOR chromocenters. The process is accompanied by a decrease in rDNA methylation and in dimethylation at histone H3K9. Here we report the same subnuclear features in Cvi-0 with respect to DNA methylation and H3K9 methylation. In addition, reduced DNA methylation has been demonstrated specifically at the rDNA loci in Cvi-0 by Riddle and Richards [35]. This suggests that *HDA6* expression or function in Cvi-0 is aberrant. However, we only identified base substitutions in small 13bp repeat sequences of the 5′-UTR region of *HDA6* in Cvi-0. It is known from yeast and animals that 5′-UTR sequences may affect protein translation [61]–[63]. For example, in some forms of breast cancer, the human *BRCA1* gene generates two isoforms due to translation from a first AUG codon and a second, in-frame [64]. If this accounts for the HDA6 gene in Cvi-0, then the sequence predicts a truncated polypeptide lacking the first 39 amino acids. This region is essential for the catalytic function of HDA6. A substitution at position 16 generated the *hda6 sil1* mutation [56]. Therefore, a truncated polypeptide lacking this N-terminal region would result in a similar (low chromatin compaction) phenotype.

In contrast, the *hda6-sil1* allele in the heterozygote L*er* x *hda6* is not dominant and the NOR chromocenter phenotype of both parents is inherited giving rise to an intermediate phenotype for total chromocenter size (see Figure 9 and Figure S1). Apparently, the presence of a functional L*er*-derived HDA6 in this heterozygote cannot restore the size of the *hda6-*derived NOR chromocenters. This suggests an epigenetic imprinting factor controlling NOR chromocenter size. This factor may involve DNA methylation, since a similar situation has been reported in the heterozygous methyltransferase mutant, *met1^+/−^* [15],[18]. Moreover, HDA6 has been shown to be mechanistically linked to methylation of repetitive DNA [15],[18],[53]. In fact, Cvi-0 and HDA6 mutants and knockouts have reduced DNA methylation in NOR genes [35],[56],[59],[60].

In summary, the analysis of natural variation among *Arabidopsis* accessions revealed a novel link between chromatin organization and light-intensity. In particular, our data implicate novel roles for the photoreceptor PHYB as positive controller of chromatin organization and for the histone modifier HDA6 in the light signaling pathway towards chromatin compaction. We propose that polymorphisms in the alleles of these genes contribute to natural variation in the nuclear phenotypes among accessions and may function in acclimation to altered light environments on the accessions collection sites.

## Materials and Methods

### Growth conditions and treatments

*Arabidopsis thaliana* accessions (Table S1), *phyB5* (N69), L*er* (NW20), Se-0 (N1502), Ts-1 (N1552), Sf-1 (N1512) were obtained from *Nottingham Arabidopsis Stock Centre (NASC)* or the *Sendai Arabidopsis Seed Stock Center (SASSC)*, Miyagi University of Education, Japan). Moss and Stange [65], Lan-0, the RILs [31] and NILs [33],[34] were provided by M. Koornneef (Wageningen University, the Netherlands). The *phyB9* and 35S::*PHYB* lines [41] were provided by J. Maloof (UC Davis, CA, USA). The *hda6 sil1/not* [36] seeds were obtained from Ian Furner (University of Cambridge, UK).

Plants were grown as previously described [66]. Unless otherwise stated, the following growth conditions were used: 20°C, 70% (v/v) relative humidity during day and night, 9 h short-day photoperiod of 200 µmol m^−2^ s^−1^ photosynthetic active radiation (PAR). Twenty-two days old plants, at developmental stage 1.05 to 1.07 [67], were used for all experiments. For all accessions this was well before the floral transition. Reduction of the light intensities below 200 µmol m^−2^ s^−1^ was accomplished by shading the plants with spectrally neutral shade cloth. The spectral quality was checked with a LICOR-1800 spectro-radiometer (LI-COR, Lincoln, NE, USA).

### Sample preparation

Young rosette leaves were harvested 1.5 h after start of the photoperiod to exclude, if any, diurnal and circadian influences, fixed in Carnoy's solution (ethanol/acetic acid 3∶1) and stored at −20°C. Each sample consisted of two plants, except in the experiment with transgenic *35S::PHYB* lines where each sample contained plants of five individual transformants. Spread preparations were made essentially as described in [68], with a modified enzymatic cell-wall degrading mixture: 0.6% Cellulase R10 (Yakult, Tokyo, Japan), 0.25% Macerozyme R10 (Duchefa, Haarlem, the Netherlands) in 10 mM citrate buffer pH 4.5. Slides were mounted in Vectashield (Vector Laboratories, Burlingame, CA, USA) with 4′,6-diamidino-2-phenylindole (DAPI; 2 µg ml^−1^) before observation. For HX calculation, 100–130 nuclei of at least two plants were analyzed. The data were compared using one-way analysis of variance (ANOVA) with Microsoft Excell 2000 (Richmond, VA, USA).

### Measure of heterochromatin index (HX) and Relative Heterochromatin Fraction (RHF)

The HX [12] was defined as the percentage of nuclei showing high content of compact chromatin (typified by L*er*; Figure 1A), represented by conspicuous chromocenters, as opposed to nuclei with less compact chromatin (typified by Cvi-0, Figure 1B). For RHF quantification, automated digital analysis of grey-scale images was carried out with *in house* developed macros in ImagePro-Plus (Media Cybernetics, Silver Spring, MD, USA). RHF, defined as the fluorescence intensity of all DAPI-stained chromocenters relative to the fluorescence of the entire nucleus, was calculated for each sample as described earlier [14],[15]. Statistical analysis was performed by Two-way analysis of variance (ANOVA), with Tukey B post hoc comparisons (SPSS), to test for significant differences between genotypes.

### Geographic climate data

For details on the geographic and environmental data see Table S2. Mean annual data (of 0.5° latitude×0.5° longitude surface land area plots) were calculated from monthly averages collected over a 30 years period (1961–1990 [24]). We tested whether the obtained data set for all individual environmental variables correlated with the latitude of the collection sites (SPSS-Sofware 12.01, Chicago, IL, USA; Table S2). If so, these sets were fitted in a linear multivariate data-regression analysis model (SPSS) using the heterochromatin index (HX) as dependent factor. Environmental factors significantly correlating to the variation in HX were found by stepwise removing the least significant (p-value) variable each time, starting from the full model, until all remaining factors were significant. Of all temperature-related traits, only the mean temperature was used in the full model because of the high correlations between these traits.

### Fluorescence *In Situ* Hybridization (FISH)

Plasmid pAL1 [69] was used to detect the 180 bp centromeric tandem repeat. BAC F28D6 (GenBank accession No. AF147262) obtained from *NASC* in pBeloBAC-Kan vector was used for the detection of pericentromeric repeats. 5S rDNA was from [70], 45S rDNA probe was from [71]. FISH experiments were carried out essentially as described in [68]. The nuclei were counterstained with DAPI (2 µg ml^−1^ in Vectashield, Vector Laboratories) prior to observation.

### 5-Methylcytosine detection

Slide preparations were dried at 60°C for 30 min, treated with 10 µg ml^−1^ RNAse A (Roche, Woerden, the Netherlands) for 60 min at 37°C, rinsed 2×5 min in PBS, fixed in 1% formaldehyde, dehydrated in successive ethanol baths and air-dried. Denaturation was carried out by adding 50 µl HB50 (50% formamide in 1xSSC) and heating at 80°C for 2 min. The slides were subsequently washed in 70% ice-cold ethanol and dehydrated by successive ethanol baths. Slides were incubated for 1 h in 1% Bovine Serum Albumine (BSA) to prevent aspecific binding and washed 3×5 min in TNT (1 M Tris/HCl; pH = 8.0; 1 M NaCl, 0.5% Tween 20). Incubation with the antibody against 5-methylcytosine (Eurogentec, Seraing, Belgium; raised in Mouse, 1∶50 in 1% BSA in PBS) was carried out at 37°C for a minimum of 1 h. Detection of the antibody was performed with the same antibodies used for FISH Digoxigenin-labeled probes as described above. The nuclei were counterstained with DAPI (2 µg ml^−1^ in Vectashield, Vector Laboratories) prior to observation.

### Immunolabeling

Leaf nuclei were isolated as previously described [13]. Immunolabeling was carried out as described in [15]. Primary antibodies were Rabbit anti-dimethyl-lysine 9 of histone H3 (Ref. No. 07-441; 1∶50, Upstate, Lake Placid, NY, USA) and Mouse anti-histone (Ref. No. 1492519; 1∶100, Roche). Slides were incubated with primary antibodies overnight at 4°C. After washing steps (37°C for 30 min) with Phosphate-Buffered-Saline (PBS) the slides were detected with antibody Donkey-anti-Rabbit∼Cy3 (1∶500, Jackson Immunoresearch Laboratories, Soham, UK) for detection in the red channel, or with antibody Donkey-anti-Mouse∼FITC (1∶200, Jackson Immunoresearch Laboratories) for detection in the green channel. The nuclei were counterstained with DAPI (2 µg ml^−1^ in Vectashield, Vector Laboratories) prior to observation.

### Image acquisition and processing

Slides were examined with an Olympus BX6000 epifluorescence microscope (Olympus, Tokyo, Japan) coupled to a CCD camera (Coolsnap FX, Photometrics, Tucson, AZ, USA). After acquisition the images were processed, pseudo-colored and merged using Adobe Photoshop software (Adobe, San Jose, CA, USA).

### Quantitative trait loci (QTL) analysis

RHF was measured in 3 to 4 weeks old plants of 47 RILs of the L*er* x Cvi-0 core-population [31]. The RILs, parental lines, and the Cvi-0 x L*er* F1s were grown in a green-house in 16 h light/8 h darkness. These long-day conditions induce the potential for flowering, a trait known to be segregating in the used RIL set [5] and was chosen to synchronize this trait, thereby circumventing interference of floral related chromatin reorganization on RHF [12]. At least 26 nuclei from 2 plants per line were used for RHF measurement.

Outliers beyond the 95% confidence interval per line were removed prior to QTL-mapping. The core-marker map [31] was used for the analysis. QTL-Cartographer algorithm: Composite Interval Mapping (CIM; http://statgen.ncsu.edu/qtlcart/WQTLCart.htm) was used, employing the “forward and backward” search method (parameters used: 10 cM window; P_in_: 0.05 P_out_: 0.05). The threshold value was determined by a 1000 permutation test (95% confidence-interval).

Broad sense heritability was calculated as part of the between-line variance attributed to the total variance, using variance components of the general linear model procedure (SPSS).

NILs were grown in short-day growth chambers, under standard growth-room conditions described above since no variation in flowering in these lines was expected [34]. NILs LCN4-3, 5-16 and 5–17 have previously been described as NIL DOG5, NIL 19–2 and NIL 30–2 respectively [33],[72].

### DNA sequencing of HDA6 genomic region

The *HDA6* gene (At5g63110) and flanking regions were PCR amplified from genomic DNA extracted from Col-0, L*er*-0, Cvi-0 and the *hda6* (*sil1*/*not*) mutant using Phusion High-Fidelity DNA Polymerase (Finnzymes, Espoo, Finland). DNA sequencing of PCR-amplified fragments of the *HDA6* encoding genomic region was performed by Macrogen (Seoul, Korea). Primer pairs used are listed in Table S3. Initially, the region encoding *HDA6* was amplified using the primers HDA6.RPA/HDA6.LPA, and internal sequencing reactions were performed using primers: HDA6.LP.A, HDA6.LP.B, HDA6.LP.C, HDA6.RP.A, HDA6.RP.B, HDA6.RP.C, HDA6.RP.D, HDA6.RP.E and HDA6.RP.F. To fill gaps in the resulting *HDA6* sequence the amplification and sequencing reactions were performed using the following primer pairs: HDA6.RPA/HDA6.LP2A, HDA6.RPB/HDA6.LP2B, HDA6.RP2C/HDA6.LP2C and HDA6.RP2D/HDA6.LP2D. The *HDA6* gene was sequenced from three independent plants from L*er*-0, Cvi-0 and *hda6* (*sil1*/*not*)). Sequences are deposited in GenBank data library under accession numbers: EU502909 (*HDA6* L*er*-0), EU502910 (*hda6* Cvi-0), EU502911 (*hda6* from *sil1*/*not* mutant).

## Supporting Information

Figure S1 Localization of BAC F28D6 to chromosomes of Col-0. DAPI staining (A) and FISH image (B) of pachytene chromosomes hybridized with a pericentric BAC F28D6 probe showing signals (green) to the pericentric region of all chromosomes in Col-0. Arrows depict centromeres. Red signal indicates euchromatic BAC T1J1 in chromosome arm 4S. NOR, nucleolar organizing region.
(0.03 MB PDF)

Figure S2 The low chromatin compaction of Cvi-0 is dominant in heterozygotes. Chromatin compaction (RHF) of *phyB5*, *hda6* (*sil1/not*) and F_1_ progeny from a cross between *hda6* and *phyb5* (gray) and the NIL-parents (black). Error bars represent standard errors. n = 13 to 40; No overlapping letters indicate a significant difference (p<0.05). Where applicable, crosses were checked with the SSLP markers polymorphic between L*er* and Cvi-0; NGA128 and NGA162 [73] with a standard PCR procedure.
(0.03 MB PDF)

Figure S3 High light intensity rescues the formation of NOR chromocenters in Cvi-0. Distribution of size per individual chromocenter in Arbitrary Pixel Units (AU) of Cvi-0 plants grown under normal (200 µmol m^−2^ s^−1^) and high light (600 µmol m^−2^ s^−1^) conditions. The highest class for both distributions is defined on a 10% cutoff.
(0.03 MB PDF)

Figure S4 The Cvi-0 NOR phenotype is dominant in crosses. Distribution of sizes per individual chromocenter in Arbitrary Pixel Units (AU) of heterozygous F_1_ plants derived from crosses between L*er* x Cvi-0 (A) and L*er* x *hda6* (B) and combined, superimposed data of the parental individuals. The highest class for both distributions is defined on a 10% cutoff.
(0.03 MB PDF)

Table S1 Names, abbreviations, stock numbers, latitudinal origin, and heterochromatin index of 21 *Arabidopsis* accessions used in this study. Plants were grown in short-day control conditions (200 µmol m^−2^ s^−1^). Standard errors never exceeded 0.13.
(0.02 MB PDF)

Table S2 Geographic and climate parameters on the collection sites of the used Arabidopsis accessions and the correlations with HX. The latitudes of the collection sites of individual accessions were taken from the Natural Variation in *Arabidopsis thaliana* (NVAT) web site: (http://dbsgap.versailles.inra.fr/vnat/) unless stated otherwise. Environmental data for all collection sites (0.5° latitude×0.5° longitude surface land area plots) of the used Arabidopsis accessions were extracted from the climate baseline data from the Intergovernmental Panel on Climate Change (IPCC) Data Distribution Centre (DCC) (http://ipcc-ddc.cru.uea.ac.uk/obs/get_30yr_means.html), using a data subtraction tool kindly provided by I. Wright (Macquarie University, Sydney, Australia). The data presented in this table are mean annual data that were calculated from monthly averages collected over a 30 years period [24]. Alt, Altitude; Long., longitude; Lat., Latitude; cloud, cloud coverage; diurnal, diurnal temperature range; Tmax, maximal temperature; Tmin, minimal temperature; Tmean, average temperature; Prec, precipitation; Irrad., Irradiation; Vapour, Vapor pressure. Wet d, wet day frequency. Corr, correlation with HX.
(0.02 MB PDF)

Table S3 DNA primers used for PCR amplification of the HDA6 gene from *Arabidopsis thaliana* genomic DNA.
(0.01 MB PDF)

Table S4 Candidate genes for the Rhf2 QTL with AGI codes, gene-model and description (The Arabidopsis Information Resource web site; www.arabidopsis.org). Genes were selected based on their annotation with the keywords: light, chromatin, or histone.
(0.02 MB PDF)

Table S5 Candidate genes for the Rhf4 QTL with AGI codes, gene-model and description (The Arabidopsis Information Resource web site; www.arabidopsis.org). Genes were selected based on their annotation with the keywords: light, chromatin, or histone.
(0.02 MB PDF)

Table S6 Candidate genes for the Rhf5 QTL with AGI codes, gene-model and description (The Arabidopsis Information Resource web site; www.arabidopsis.org). Genes were selected based on their annotation with the keywords: light, chromatin, or histone.
(0.02 MB PDF)
