# Supplementary figures and images for: PHYTOCHROME B and HISTONE DEACETYLASE 6 Control Light-Induced Chromatin Compaction in Arabidopsis thaliana
Source: PLoS Genet. 2009 Sep 4;5(9):e1000638. doi: 10.1371/journal.pgen.1000638 (PMC2728481; doi:10.1371/journal.pgen.1000638)

**Figure S1**

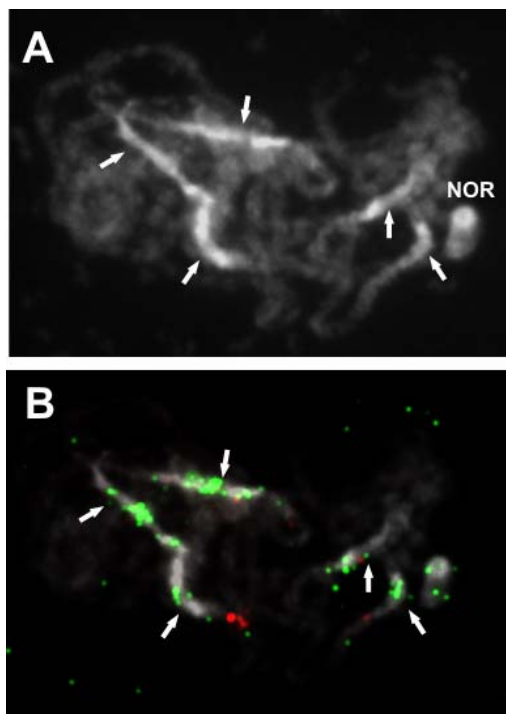

Supplement: Figure S1 — Localization of BAC F28D6 to chromosomes of Col-0. DAPI staining (A) and FISH image (B) of pachytene chromosomes hybridized with a pericentric BAC F28D6 probe showing signals (green) to the pericentric region of all chromosomes in Col-0. Arrows depict centromeres. Red signal indicates euchromatic BAC T1J1 in chromosome arm 4S. NOR, nucleolar organizing region. (0.03 MB PDF) [file pgen.1000638.s001.pdf]

Figure S2

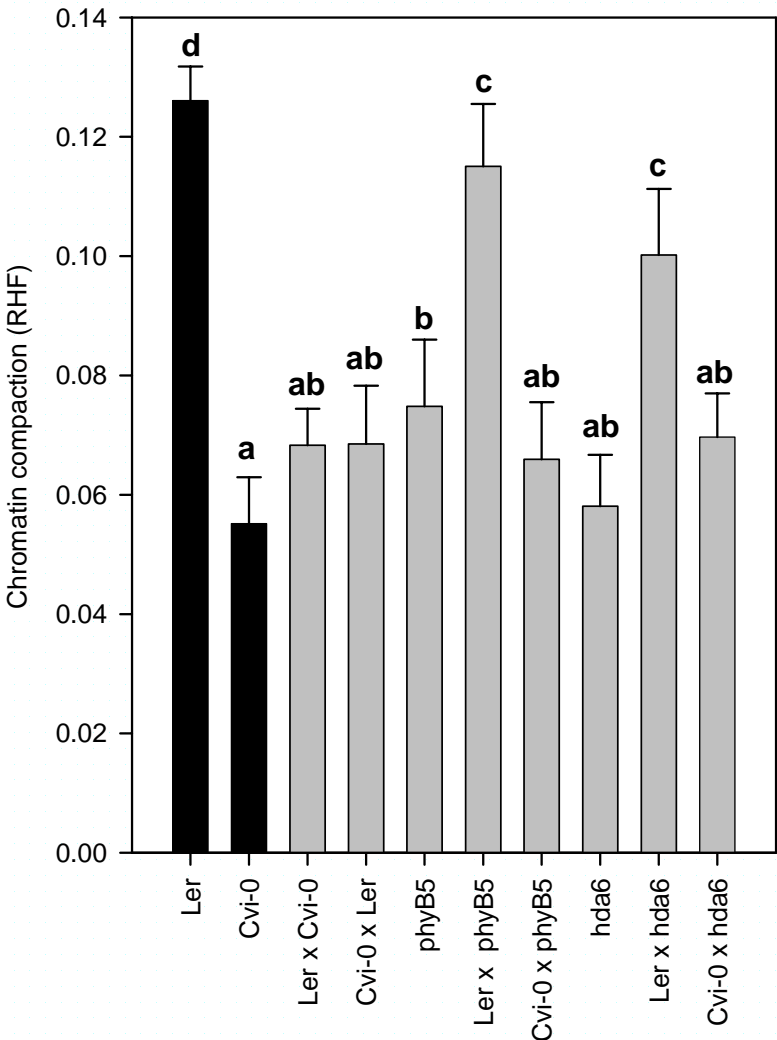

Supplement: Figure S2 — The low chromatin compaction of Cvi-0 is dominant in heterozygotes. Chromatin compaction (RHF) of phyB5, hda6 (sil1/not) and F1 progeny from a cross between hda6 and phyb5 (gray) and the NIL-parents (black). Error bars represent standard errors. n = 13 to 40; No overlapping letters indicate a significant difference (p<0.05). Where applicable, crosses were checked with the SSLP markers polymorphic between Ler and Cvi-0; NGA128 and NGA162 [73] with a standard PCR procedure. (0.03 MB PDF) [file pgen.1000638.s002.pdf]

**Figure S3**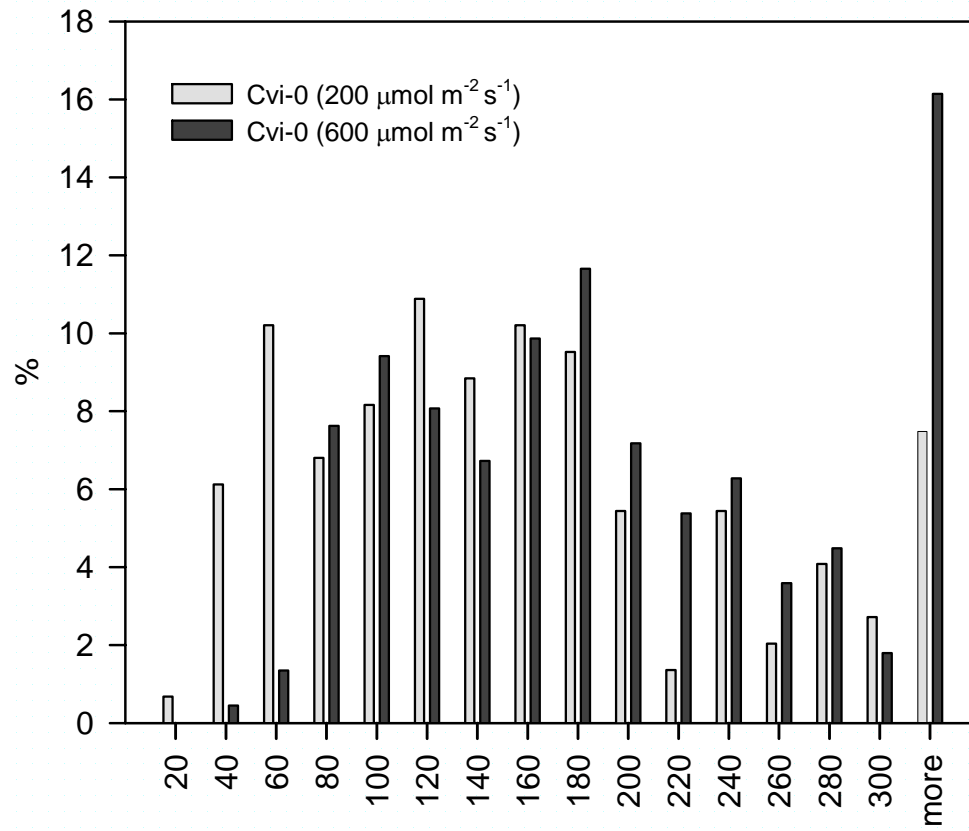

Supplement: Figure S3 — High light intensity rescues the formation of NOR chromocenters in Cvi-0. Distribution of size per individual chromocenter in Arbitrary Pixel Units (AU) of Cvi-0 plants grown under normal (200 µmol m−2 s−1) and high light (600 µmol m−2 s−1) conditions. The highest class for both distributions is defined on a 10% cutoff. (0.03 MB PDF) [file pgen.1000638.s003.pdf]

Figure S4

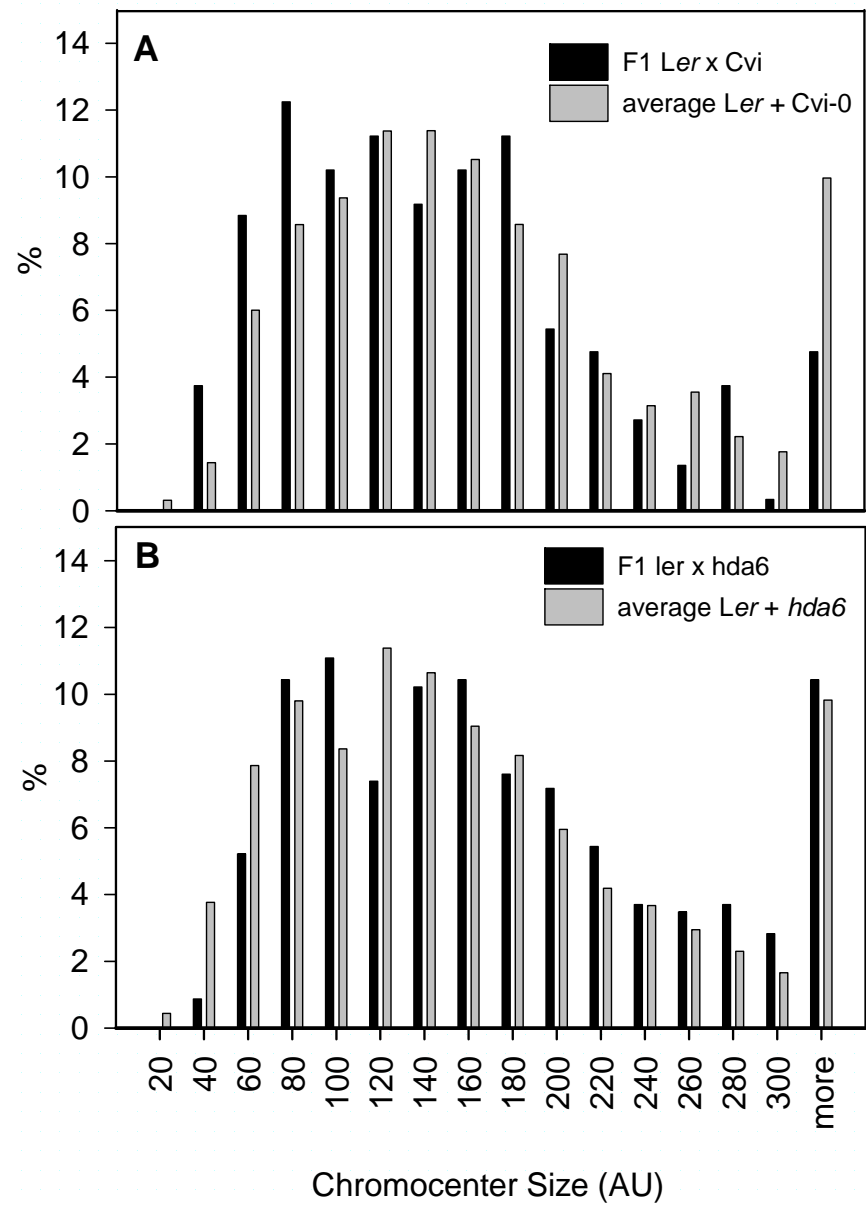

Supplement: Figure S4 — The Cvi-0 NOR phenotype is dominant in crosses. Distribution of sizes per individual chromocenter in Arbitrary Pixel Units (AU) of heterozygous F1 plants derived from crosses between Ler x Cvi-0 (A) and Ler x hda6 (B) and combined, superimposed data of the parental individuals. The highest class for both distributions is defined on a 10% cutoff. (0.03 MB PDF) [file pgen.1000638.s004.pdf]
