# Supplementary material for: PHYTOCHROME B and HISTONE DEACETYLASE 6 Control Light-Induced Chromatin Compaction in Arabidopsis thaliana
Source: PLoS Genet. 2009 Sep 4;5(9):e1000638. doi: 10.1371/journal.pgen.1000638 (PMC2728481; doi:10.1371/journal.pgen.1000638)
Supplement: Table S1 — Names, abbreviations, stock numbers, latitudinal origin, and heterochromatin index of 21 Arabidopsis accessions used in this study. Plants were grown in short-day control conditions (200 µmol m−2 s−1). Standard errors never exceeded 0.13. (0.02 MB PDF) [file pgen.1000638.s005.pdf]

Table S1

Name	Abb.	Stock no.	HX
Bensheim-0	Be-0	N964	0.80
C24	C24	N906	0.76
Canary Islands-0	Can-0	N1064	0.49
Cape Verde Island-0	Cvi-0	N902	0.19
Chisdra	Chi-1	N1074	0.83
Columbia-0	Col-0	N1092	0.84
Helsinki-1	Hel-1	N1222	0.85
Hiroshima-1	Hir-1	JSW102	0.68
Moss			0.76
Kärnten-0	Kä-0	N1266	0.76
Kashmir-2	Kas-2	N903	0.90
Knox-10	Knox-10	N22566	0.83
Kondana-Tady	Kond	N916	0.92
Landsberg-0	Lan-0	N1298	0.85
Martuba / Cyrenaika-0	Mt-0	N1380	0.67
Niederzenz-1	Nd-1	N1636	0.91
Pakistan-1	Pak-1	JW105	0.74
RLD	RLD-1	N913	0.87
Shahdara	Shah	N929	0.87
Stange			0.90
Wassilewskija-2	Ws-2	N915	0.78

Seeds were obtained from the *Nottingham Arabidopsis Stock Centre (NASC),* (http://arabidopsis.info/), except accession: Hir-1 and Pak-1, which were obtained from the *Sendai Arabidopsis Seed Stock Center (SASSC)* Miyagi University of Education, Japan (http://www.brc.riken.jp/lab/epd/Eng/index.shtml). Accessions; Lan-0, Moss and Stange [65] were a gift from M. Koornneef (Wageningen University. the Netherlands).
