# Supplementary material for: PHYTOCHROME B and HISTONE DEACETYLASE 6 Control Light-Induced Chromatin Compaction in Arabidopsis thaliana
Source: PLoS Genet. 2009 Sep 4;5(9):e1000638. doi: 10.1371/journal.pgen.1000638 (PMC2728481; doi:10.1371/journal.pgen.1000638)
Supplement: Table S2 — Geographic and climate parameters on the collection sites of the used Arabidopsis accessions and the correlations with HX. The latitudes of the collection sites of individual accessions were taken from the Natural Variation in Arabidopsis thaliana (NVAT) web site: (http://dbsgap.versailles.inra.fr/vnat/) unless stated otherwise. Environmental data for all collection sites (0.5° latitude×0.5° longitude surface land area plots) of the used Arabidopsis accessions were extracted from the climate baseline data from the Intergovernmental Panel on Climate Change (IPCC) Data Distribution Centre (DCC) (http://ipcc-ddc.cru.uea.ac.uk/obs/get_30yr_means.html), using a data subtraction tool kindly provided by I. Wright (Macquarie University, Sydney, Australia). The data presented in this table are mean annual data that were calculated from monthly averages collected over a 30 years period [24]. Alt, Altitude; Long., longitude; Lat., Latitude; cloud, cloud coverage; diurnal, diurnal temperature range; Tmax, maximal temperature; Tmin, minimal temperature; Tmean, average temperature; Prec, precipitation; Irrad., Irradiation; Vapour, Vapor pressure. Wet d, wet day frequency. Corr, correlation with HX. (0.02 MB PDF) [file pgen.1000638.s006.pdf]

Table S2

	Alt<br>m	Long<br>^{o}W	Lat<br>^{o}N	cloud<br>%	diurnal<br>^{o}C	Tmax<br>^{o}C	Tmean<br>^{o}C	Tmin<br>^{o}C	prec<br>mm day^{-1}	Irrad [f]<br>µmol.m^{-2} s^{-1}	vapour<br>hPa	wet d<br>day
**Be-0**	150	8.37	49.41	71.58	8.83	14.58	10.15	5.75	1.89	507.65	9.83	14.46
**C24** [d]	150	8.24	40.20	47.75	7.53	19.43	15.65	11.91	1.92	711.00	13.18	8.42
**Can-0** [a]	1250	-15.3	28.00	53.83	6.65	19.63	16.28	12.98	1.15	756.71	12.97	8.97
**Chi-1**	150	35	54.00	69.08	7.99	9.34	5.34	1.34	1.73	500.79	8.13	15.05
**Col-0**	50	15.15	52.44	68.58	8.11	12.82	8.73	4.69	1.50	501.94	9.39	13.70
**Cvi-0** [a]	1200	-24.4	14.90	34.71	14.77	34.76	27.35	19.99	1.16	982.17	18.83	6.30
**Hel-1** [a]	50	25	60.00	68.54	7.70	8.41	4.56	0.70	1.79	447.99	7.77	15.18
**Hir-1** [a]	50	33.2	34.50	40.67	10.10	23.40	18.33	13.30	1.41	824.89	13.49	6.21
**Ka-0**	950	14.31	46.25	60.17	8.18	11.97	7.85	3.77	4.62	593.34	8.98	13.89
**Kas-1**	1550	77	34.00	51.58	11.67	6.35	5.08	-5.33	1.25	752.14	0.58	5.23
**Knox-1**		-88.63	41.58	63.92	11.23	14.18	8.54	2.94	2.45	602.10	9.55	10.52
**Kond**	1050	68.5	38.48	49.00	13.79	21.47	14.55	7.67	1.22	739.96	9.33	7.16
**La-0** [e]	50	15.5	52.50	67.33	8.18	12.83	8.72	4.63	1.46	508.03	9.42	13.66
**Moss** [c]	70	10.42	59.28	64.33	7.23	9.88	6.23	1.53	2.47	473.00	8.15	15.00
**Mt-0**	150	22.46	32.34	34.17	11.38	23.99	18.27	12.61	0.97	901.43	14.12	4.62
**Nd-1**	250	8.02	50.28	73.58	7.93	12.69	8.70	4.74	1.95	481.76	9.17	15.04
**Pak-1** [b]		73.4	33.90	38.08	13.25	26.33	19.68	13.08	3.07	832.50	13.65	8.45
**RLD-1**	50	5.3	52.15	76.25	7.85	13.20	9.26	5.34	2.19	447.48	10.21	15.92
**Shah**	3400	71	37.00	50.83	10.47	7.21	1.94	-3.28	1.58	738.06	0.28	8.53
**Stange** [c]	120	11.14	60.30	61.33	8.46	8.38	4.14	-0.08	2.00	449.00	7.22	15.37
**Ws-2**	150	30.38	52.13	67.08	8.62	11.19	6.88	2.57	1.66	522.50	8.63	13.71
**Corr** [g]				0.82<br>***	-0.68<br>***	-0.75<br>***	-0.76<br>***	-0.64<br>**	0.26<br>n.s.	-0.95<br>***	-0.43<br>n.s.	0.88<br>***

a No climate data was available on the 0.5° latitude × 0.5° plots for these locations. Therefore, average values of surrounding plots were used.

b Geographic characteristics were obtained from [74].

c Geographic characteristics were obtained [65].

d C24 most likely originates from the Portuguese accession; Coimbra [75]. We used the Coimbra geographic characteristics (NVAT) in our analysis.

e We used the original Landsberg wild-type accession in our analysis which did not differ in HX and RHF from mutant; Landsberg *erecta.*

f Total irradiation was converted from W.m^{-2} to µmol m^{-2} s^{-1} as average for 24 hours using the conversion factor 4.57 for the PAR spectrum (400-700 nm) as described in [76].

g Correlation*;* 2-tailed Pearson correlation coefficient n.s. non-significant; * $p<0.05$; ** $p<0.01$; *** $p<0.001$; n.s: non significant.
