# Supplementary material for: PHYTOCHROME B and HISTONE DEACETYLASE 6 Control Light-Induced Chromatin Compaction in Arabidopsis thaliana
Source: PLoS Genet. 2009 Sep 4;5(9):e1000638. doi: 10.1371/journal.pgen.1000638 (PMC2728481; doi:10.1371/journal.pgen.1000638)
Supplement: Table S3 — DNA primers used for PCR amplification of the HDA6 gene from Arabidopsis thaliana genomic DNA. (0.01 MB PDF) [file pgen.1000638.s007.pdf]

Table S3

Primer name	Primer Sequence (5' to 3')
HDA6.LP.A	ATTGCAATCGGTTTTCTTCG
HDA6.LP.B	CCGACATTATCATATGGCAAAA
HDA6.LP.C	ACTCCATCTCCATGGTGGAC
HDA6.LP2A	CAGAGAGATGCCGCTTTCG
HDA6.LP2B	CAGAAGAACATGAAGAAGTTC
HDA6.LP2C	GCAACTGAAGTACAATGACC
HDA6.LP2D	GTTTAGTAACAGCTTCAATGG
HDA6.RP.A	GCTGATTTCGCTGCTTCTTC
HDA6.RP.B	AGCTGTGCCCACAACTCCTA
HDA6.RP.C	GGGCTTCACCATGCTAAGAA
HDA6.RP.D	GTCCACCATGGAGATGGAGT
HDA6.RP.E	GGCCCAGATTATACGCTTCA
HDA6.RP.F	CTGGAGTGGAACTGCGACTT
HDA6.RP2C	GAGAATGGTTTTCTACATTGC
HDA6.RP2D	GGAACACGTTGCTGGAAC
