# Supplementary material for: PHYTOCHROME B and HISTONE DEACETYLASE 6 Control Light-Induced Chromatin Compaction in Arabidopsis thaliana
Source: PLoS Genet. 2009 Sep 4;5(9):e1000638. doi: 10.1371/journal.pgen.1000638 (PMC2728481; doi:10.1371/journal.pgen.1000638)
Supplement: Table S4 — Candidate genes for the Rhf2 QTL with AGI codes, gene-model and description (The Arabidopsis Information Resource web site; www.arabidopsis.org). Genes were selected based on their annotation with the keywords: light, chromatin, or histone. (0.02 MB PDF) [file pgen.1000638.s008.pdf]

Table S4

AGI code	Associated gene model	Description
AT2G18790	PHYB	Red/far-red photoreceptor involved in the regulation of de-etiolation. Exists in two inter-convertible forms: Pr and Pfr (active). Involved in the light-promotion of seed germination and in the shade avoidance response.
AT2G14820		phototropic-responsive NPH3 family protein; similar to phototropic-responsive NPH3 family protein [Arabidopsis thaliana] (TAIR:AT5G67440.1)
AT2G19230		leucine-rich repeat protein kinase. putative; similar to FRK1 (FLG22-INDUCED RECEPTOR-LIKE KINASE 1). kinase [Arabidopsis thaliana] (TAIR:AT2G19190.1); similar to light repressible receptor protein kinase [Arabidopsis thaliana] (TAIR:AT4G29990.1); similar to leucine-rich repeat protein kinase. putative [Arabidopsis thaliana] (TAIR:AT2G19210.1)
AT2G19310		similar to HSP18.2 (HEAT SHOCK PROTEIN 18.2) [Arabidopsis thaliana] (TAIR:AT5G59720.1);
AT2G20180	PIL5	PIL5 (PHYTOCHROME INTERACTING FACTOR 3-LIKE 5); transcription factor; similar to transcription regulator [Arabidopsis thaliana] (TAIR:AT4G28811.1);
AT2G19210		leucine-rich repeat protein kinase. putative; similar to FRK1 (FLG22-INDUCED RECEPTOR-LIKE KINASE 1). kinase [Arabidopsis thaliana] (TAIR:AT2G19190.1); similar to light repressible receptor protein kinase [Arabidopsis thaliana] (TAIR:AT4G29990.1); similar to leucine-rich repeat protein kinase. putative [Arabidopsis thaliana] (TAIR:AT2G19230.1);
AT2G18915	LKP2	encodes a member of F-box proteins that includes two other proteins in Arabidopsis (ZTL and FKF1). These proteins contain a unique structure containing a PAS domain at their N-terminus. an F-box motif. and 6 kelch repeats at their C-terminus. Overexpression results in arrhythmic phenotypes for a number of circadian clock outputs in both constant light and constant darkness. long hypocotyls under multiple fluences of both red and blue light. and a loss of photoperiodic control of flowering time. Although this the expression of this gene itself is not regulated by circadian clock. it physically interacts with Dof transcription factors that are transcriptionally regulated by circadian rhythm. LKP2 interacts with Di19. CO/COL family proteins.
AT2G17840	ERD7	Identified as drought-inducible gene by differential hybridization. Upregulated by high light. drought. cold and salt stress determined by microarray analysis.
AT2G19540		transducin family protein / WD-40 repeat family protein; similar to MSI2 (NUCLEOSOME/CHROMATIN ASSEMBLY FACTOR GROUP C 2) [Arabidopsis thaliana] (TAIR:AT2G16780.1)
AT2G18760	CHR8	CHR8 (chromatin remodeling 8); ATP binding / DNA binding / helicase; similar to CHR24 (chromatin remodeling 24). ATP binding / DNA binding / helicase [Arabidopsis thaliana] (TAIR:AT5G63950.1)
AT2G17560	HMGB4	Encodes a protein belonging to the subgroup of HMGB (high mobility group B) proteins that have a distinctive DNA-binding motif. the HMG-box domain. The motif confers non-sequence specific interaction with linear DNA and structure-specific binding to distorted DNA sites. The HMGB proteins are involved in the assembly of nucleoprotein complexes. Can be phosphorylated by CK2alpha.
AT2G16390	DRD1	Putative chromatin remodeling protein. member of a plant-specific subfamily of SWI2/SNF2-like proteins. Mutations nearly eliminate non-CpG methylation at a target promoter but do not affect rDNA or centromere methylation. Cooperates with PolIVb to facilitate RNA-directed de novo methylation and silencing of homologous DNA. Endogenous targets include intergenic regions near retrotransposon LTRs or short RNA encoding sequences that might epigenetically regulate adjacent genes. May be used to establish a basal yet reversible level of silencing in euchromatin.
AT2G19640		SET domain-containing protein; Identical to Histone-lysine N-methyltransferase ASHR2 (EC 2.1.1.43) (ASH1-related protein 2) (Protein SET DOMAIN GROUP 39) (ASHR2) [Arabidopsis Thaliana] (GB:Q9ZUM9;GB:Q84WB9;GB:Q94CD2); similar to unknown protein [Arabidopsis thaliana] (TAIR:AT5G06620.1)
AT2G13570		CCAAT-box binding transcription factor. putative; Identical to Nuclear transcription factor Y subunit B-7 (AtNF-YB-7) (NFYB7) [Arabidopsis Thaliana] (GB:Q9SIT9); similar to CCAAT-box binding transcription factor subunit B (NF-YB) (HAP3) (AHAP3) family [Arabidopsis thaliana] (TAIR:AT4G14540.1)
AT2G18050	HIS1-3	encodes a structurally divergent linker histone whose gene expression is induced by dehydration and ABA.
