# Supplementary material for: PHYTOCHROME B and HISTONE DEACETYLASE 6 Control Light-Induced Chromatin Compaction in Arabidopsis thaliana
Source: PLoS Genet. 2009 Sep 4;5(9):e1000638. doi: 10.1371/journal.pgen.1000638 (PMC2728481; doi:10.1371/journal.pgen.1000638)
Supplement: Table S5 — Candidate genes for the Rhf4 QTL with AGI codes, gene-model and description (The Arabidopsis Information Resource web site; www.arabidopsis.org). Genes were selected based on their annotation with the keywords: light, chromatin, or histone. (0.02 MB PDF) [file pgen.1000638.s009.pdf]

## Table S5

| AGI code | Associated gene model | Description |
|---|---|---|
| AT4G08920 | CRY1 | Encodes a flavin-type blue-light photoreceptor with ATP binding and autophosphorylation activity. The photoreceptor may be involved in electron transport. Mutant phenotype displays a blue light-dependent inhibition of hypocotyl elongation. Photoreceptor activity requires light-induced homodimerisation of the N-terminal CNT1 domains of CRY1.Involved in blue-light induced stomatal opening. The C-terminal domain of the protein undergoes a light dependent conformational change. Also involved in response to circadian rhythm. Mutants exhibit long hypocotyl under blue light and is out of phase in their response to circadian rhythm. |
| AT4G10340 | LHCB5 | photosystem II encoding the light-harvesting chlorophyll a/b binding protein CP26 of the antenna system of the photosynthetic apparatus |
| AT4G10840 | | tetratricopeptide repeat (TPR)-containing protein; similar to kinesin light chain-related [Arabidopsis thaliana] (TAIR:AT4G10840.1); |
| AT4G11110 | SPA2 | Encodes a member of the SPA (suppressor of phyA-105) protein family (SPA1-SPA4). SPA proteins contain an N-terminal serine/threonine kinase-like motif followed by a coiled-coil structure and a C-terminal WD-repeat domain. SPA proteins function redundantly in suppressing photomorphogenesis in dark- and light-grown seedlings. SPA2 primarily regulates seedling development in darkness and has little function in light-grown seedlings or adult plants. |
| AT4G12400 | | binding; similar to stress-inducible protein, putative [Arabidopsis thaliana] (TAIR:AT1G12270.1); similar to stress-inducible protein, putative [Arabidopsis thaliana] (TAIR:AT1G62740.1) |
| AT4G12800 | PHOTOSYSTEM I SUBUNIT L | Encodes subunit L of photosystem I reaction center. |
| AT4G13790 | | auxin-responsive protein, putative; similar to SAUR_AC1 (SMALL AUXIN UP RNA 1 FROM ARABIDOPSIS THALIANA ECOTYPE COLUMBIA) [Arabidopsis thaliana] (TAIR:AT4G38850.1); |
| AT4G14110 | COP9 | Represses photomorphogenesis and induces skotomorphogenesis in the dark. A component of the COP9 signalosome complex. |
| AT4G14690 | ELIP2 | Encodes an early light-induced protein. ELIPs are thought not to be directly involved in the synthesis and assembly of specific photosynthetic complexes, but rather affect the biogenesis of all chlorophyll-binding complexes. A study (PMID 17553115) has shown that the chlorophyll synthesis pathway was downregulated as a result of constitutive ELIP2 expression, leading to decreased chlorophyll availability for the assembly of pigment-binding proteins for photosynthesis. |
| AT4G15090 | FAR1 | Encodes a nuclear localized protein involved in far red light response signaling. Loss of function mutants are defective in far red light responses. Interacts with homologous gene FHY3. |
| AT4G15530 | PPDK | PPDK (PYRUVATE ORTHOPHOSPHATE DIKINASE); |
| AT4G15930 | | dynein light chain, putative; similar to dynein light chain, putative [Arabidopsis thaliana] (TAIR:AT5G20110.1) |
| AT4G16146 | | similar to unknown protein [Arabidopsis thaliana] (TAIR:AT1G69510.2) |
| AT4G16250 | PHYD | Encodes a phytochrome photoreceptor with a function similar to that of phyB that absorbs the red/far-red part of the light spectrum and is involved in light responses. It cannot compensate for phyB loss in Arabidopsis but can substitute for tobacco phyB in vivo. |
| AT4G16520 | ATG8F | ATG8F (AUTOPHAGY 8F); microtubule binding; similar to AtATG8e (AUTOPHAGY 8E), microtubule binding [Arabidopsis thaliana] (TAIR:AT2G45170.2); |
| AT4G10180 | DET1 | Encodes a nuclear-localized protein that acts as a repressor of photomorphogenesis and may be involved in chromatin remodeling. |
| AT4G10670 | GTC2 | Homologous to yeast SPT16, a general chromatin factor required for transcription |
| AT4G10710 | SPT16 | encodes a component of the FAcilitates Chromatin Transcription (FACT) complex, SPT16 |
| AT4G13460 | SUVH9 | Encodes a SU(VAR)3-9 homolog, a SET domain protein. Known SET domain proteins are involved in epigenetic control of gene expression. There are 10 SUVH genes in Arabidopsis and this subfamily of the SET proteins have an additional conserved RING finger motif called YDG domain. |
| AT4G13940 | HOG1 | Encodes a S-adenosyl-L-homocysteine hydrolase required for DNA methylation-dependent gene silencing. |
| AT4G16845 | VRN2 | The VERNALIZATION2 (VRN2) gene mediates vernalization and encodes a nuclear-localized zinc finger protein with similarity to Polycomb group (PcG) proteins of plants and animals. In wild-type Arabidopsis, vernalization results in the stable reduction of the levels of the floral repressor FLC. In vrn2 mutants, FLC expression is downregulated normally in response to vernalization, but instead of remaining low, FLC mRNA levels increase when plants are returned to normal temperatures. VRN2 maintains FLC repression after a cold treatment, serving as a mechanism for the cellular memory of vernalization. Required for complete repression of FLC. Required for the methylation of histone H3 |
| AT4G15180 | | SET domain-containing protein; similar to EFS (EARLY FLOWERING IN SHORT DAYS) [Arabidopsis thaliana] (TAIR:AT1G77300.2) |
| AT4G13570 | HTA4 | Encodes HTA4, a histone H2A protein. |
