# Supplementary material for: PHYTOCHROME B and HISTONE DEACETYLASE 6 Control Light-Induced Chromatin Compaction in Arabidopsis thaliana
Source: PLoS Genet. 2009 Sep 4;5(9):e1000638. doi: 10.1371/journal.pgen.1000638 (PMC2728481; doi:10.1371/journal.pgen.1000638)
Supplement: Table S6 — Candidate genes for the Rhf5 QTL with AGI codes, gene-model and description (The Arabidopsis Information Resource web site; www.arabidopsis.org). Genes were selected based on their annotation with the keywords: light, chromatin, or histone. (0.02 MB PDF) [file pgen.1000638.s010.pdf]

Table S6

AGI code	Associated gene model	Description
AT5G63310	NDPK2	Maintains intracellular dNTP levels except ATP. Plays a role in response to oxidative stress and UV. Involved in phytochrome-mediated light signaling. Participates in auxin-regulated processes. partly through the modulation of auxin transport. H-bonding with His-197 inside the nucleotide-binding pocket is critical for NDPK2 functioning.
AT5G63570	GSA1	Encodes a protein with homology to glutamate-1-semialdehyde 2.1-aminomutase catalyzing the conversion of glutamate-1-semialdehyde (GSA) into 5-amino levulinate. The expression of this gene was demonstrated to be light-induced.
AT5G63870	PP7	Encodes a nuclear localized serine/threonine phosphatase that appears to be regulated by redox activity and is a positive regulator of cryptochrome mediated blue light signalling.
AT5G64330	NPH3	Involved in blue light response signaling pathway; interacts with the blue light photoreceptor NPH1.Null mutations abolish phototrophic responses of etiolated seedlings to low fluence blue light. Protein contains multiple protein-protein interaction domains.
AT5G64740	CESA6	Encodes a cellulase synthase. Mutants are defective in hypocotyl elongation dark-grown plants. Normal hypocotyl elongation is restored in plants grown in white. blue or red light.
AT5G66560		phototropic-responsive NPH3 family protein; similar to phototropic-responsive NPH3 family protein [Arabidopsis thaliana] (TAIR:AT3G50840.1)
AT5G66570	PSBO-1	Encodes a protein which is an extrinsic subunit of photosystem II and which has been proposed to play a central role in stabilization of the catalytic manganese cluster. In *Arabidopsis thaliana* the PsbO proteins are encoded by two genes: *psbO1* and *psbO2*. PsbO1 is the major isoform in the wild-type.
AT5G67030	ABA1	Encodes a single copy zeaxanthin epoxidase gene that functions in first step of the biosynthesis of the abiotic stress hormone abscisic acid (ABA). Mutants in this gene are unable to express female sterility in response to beta-aminobutyric acid. as wild type plants do.
AT5G67385		signal transducer; similar to phototropic-responsive protein. putative [Arabidopsis thaliana] (TAIR:AT3G49970.1)
AT5G63110	HDA6	RPD3-like histone deacetylase. HDA6 mutations specifically increase the expression of auxin-responsive transgenes. suggesting a role in transgene silencing
AT5G63470	MLE2.10	CCAAT-box binding transcription factor Hap5a. putative; Identical to Nuclear transcription factor Y subunit C-4 (AtNF-YC-4) (NFYC4) [Arabidopsis Thaliana] (GB:Q9FMV5); similar to HAP5A (Heme activator protein (yeast) homolog 5A). DNA binding / transcription factor [Arabidopsis thaliana] (TAIR:AT3G48590.1)
AT5G63860	UVR8	UV-B-specific signaling component that orchestrates expression of a range of genes with vital UV-protective functions. Located in the nucleus and the cytosol. Associates with chromatin via histones.
AT5G63880	MGI19.8	SNF7 family protein; similar to SNF7-related [Arabidopsis thaliana] (TAIR:AT5G09260.1);
AT5G63950	CHR24	CHR24 (chromatin remodeling 24); ATP binding / DNA binding / helicase; similar to CHR8 (chromatin remodeling 8). ATP binding / DNA binding / helicase [Arabidopsis thaliana] (TAIR:AT2G18760.1)
AT5G64610	MUB3.13	histone acetyltransferase. putative; Identical to Probable MYST-like histone acetyltransferase 1 (EC 2.3.1.48) (HAG4) [Arabidopsis Thaliana] (GB:Q9FLF7); similar to histone acetyltransferase. putative [Arabidopsis thaliana] (TAIR:AT5G09740.1
AT5G64630	FAS2	Chromatin Assembly Factor-1 (CAF-1) p60 subunit. Involved in organization of the shoot and root apical meristems.
AT5G65350	MNA5.8	histone H3; Identical to Histone H3-like 5 [Arabidopsis Thaliana] (GB:Q9FKQ3); similar to histone H3 [Arabidopsis thaliana] (TAIR:AT5G10390.1); similar to histone H3 [Arabidopsis thaliana] (TAIR:AT5G65360.1); similar to histone H3 [Arabidopsis thaliana] (TAIR:AT5G10400.1)
AT5G66750	DDM1	Protein is similar to SWI2/SNF2 chromatin remodeling proteins. Involved in gene silencing and maintenance of DNA methylation and histone methylation.
AT5G67580	ATTRB2	ATTRB2/TRB2 (TELOMERE REPEAT BINDING FACTOR 2); DNA binding / transcription factor; similar to ATTRB3/TRB3 (TELOMERE REPEAT BINDING FACTOR 1). DNA binding / transcription factor [Arabidopsis thaliana] (TAIR:AT3G49850.1)
